# Exploration of the Interplay Between Trait Emotional Intelligence Dimensions and Creative Potential in Childhood

**DOI:** 10.3390/bs16061016

**Published:** 2026-06-17

**Authors:** Stefano Rini, Zeynep Özal, Giovanni Emanuele Corazza, Giacomo Mancini, Sergio Agnoli

**Affiliations:** 1 Department of Electrical, Electronic, and Information Engineering “Guglielmo Marconi”, Marconi Institute for Creativity, University of Bologna, 40126 Bologna, Italy; stefano.rini@unibo.it (S.R.); giovanni.corazza@unibo.it (G.E.C.); 2 Department of Education Sciences “G.M. Bertin”, Alma Mater Studiorum, University of Bologna, 40126 Bologna, Italy; zeynep.ozal2@unibo.it; 3 Department of Life Sciences, University of Trieste, 34127 Trieste, Italy; sergio.agnoli@units.it

**Keywords:** children, trait emotional intelligence, creativity, creative potential, EPoC

## Abstract

Trait Emotional Intelligence (Trait EI) has been increasingly linked to children’s adaptive functioning; however, its role in creative thinking remains underexplored, particularly at the level of individual facets. This exploratory study examined the relationship between trait EI dimensions and creative potential in primary school children. A sample of 344 students (165 females) aged 8–11 years completed the TEIQue-Child Form and the graphical tasks of the Evaluation of Potential Creativity (EPoC), assessing divergent and convergent thinking in both abstract and concrete domains. Multiple regression analyses tested the predictive role of trait EI facets and their interaction with age in children’s creative potential. The results indicated that Adaptability and Self-Motivation positively predicted divergent thinking, particularly in abstract tasks. Notably, the effect of Adaptability varied across grades, showing a stronger association in younger children and a progressive decrease with age, whereas Self-Motivation displayed a more stable pattern. No significant associations emerged for convergent thinking, which, however, showed a developmental trend characterized by a fourth-grade slump. Overall, the findings suggest that specific emotional self-perceptions selectively support divergent thinking during childhood and that these effects are developmentally sensitive. These results contribute to a more nuanced understanding of the relationship between Trait EI and creativity, highlighting the value of a facet-level approach and of a developmental perspective.

## 1. Introduction

Creativity is widely recognized as a fundamental developmental resource in childhood because it supports learning, problem-solving, flexible adaptation, and participation in increasingly complex educational and social contexts (Barbot et al., 2015; Alves-Oliveira et al., 2022; Corazza et al., 2021). The standard definition (Runco & Jaeger, 2012) states that creativity relies on two main requirements, originality and effectiveness, which essentially identify a creative achievement. From a broader perspective, the dynamic definition of creativity (Corazza, 2016) states that creativity requires a potential for originality and effectiveness, subsuming both creative achievement and creative inconclusiveness, taking into account the dynamic essence of the phenomenon. Creativity is indeed not only a property of products but also a dynamic, goal-directed, and context-sensitive process (Corazza & Agnoli, 2026; Mumford & McIntosh, 2017). Moreover, Walia (2019) stated that the unfolding of the creative act occurs across person, process, product, and environment; Green et al. (2024) distinguished creativity as a process from the creative quality of an outcome and defined it in terms of internally directed attention constrained by a generative goal; Dow (2022) underlined the importance of definitions that are theoretically precise and operationally useful; and more recently, Runco (2025), taking into account the current technological revolution introduced by AI, argued that contemporary definitions should also include criteria such as authenticity and intentionality when distinguishing human from artificial creativity. In childhood, this potential-oriented view is particularly relevant because creative potential is still developing and is shaped by cognitive, emotional, motivational, and contextual resources.

For empirical purposes, the present study focuses on creative potential rather than on established creative achievement. Creative potential can be understood as the set of developing resources that enable a child to produce ideas or products that may become original and effective under appropriate task and contextual conditions (Barbot et al., 2015; Lubart et al., 2011). Within this framework, divergent thinking and convergent thinking are not equated with creativity as a whole; rather, they are treated as complementary indicators of creative potential. Divergent thinking refers to the exploratory generation of multiple, varied, and potentially original responses to open-ended problems, whereas convergent thinking refers to the integration or selection of elements into a coherent and original solution (Cropley, 2006; Gabora, 2018; Runco & Acar, 2012). This distinction is especially useful in school-age samples because children may show uneven development across creative processes, domains, and task formats.

Understanding creative potential in childhood is important for developmental and educational psychology. Creative thinking supports children in moving beyond routine answers, exploring alternative perspectives, and coping with novel learning situations. These capacities are increasingly relevant in classrooms that require problem-solving, collaboration, and adaptation, but they are also developmentally sensitive: previous research has documented both increases and temporary slumps in children’s creative performance across the primary school years (Charles & Runco, 2001; Claxton et al., 2005; Gralewski et al., 2016; Kim, 2011; Runco et al., 2017). Clarifying which individual-difference variables are associated with different components of creative potential can, therefore, help to explain why children of similar age and educational exposure may differ in their ability to generate or integrate creative ideas.

One set of individual differences that may be particularly relevant concerns emotional processes. Research on emotion–cognition interactions indicates that emotions do not merely accompany thought; they shape attention, cognitive flexibility, persistence, and evaluative control (Agnoli & Corazza, 2019; De Dreu et al., 2008; Okon-Singer et al., 2015; Pessoa, 2008; Pessoa, 2017). These mechanisms are central to creative ideation, which often requires the child to tolerate ambiguity, remain engaged when a solution is not immediately available, and shift flexibly among possible meanings or representations. Emotional Intelligence (EI) has, therefore, been investigated as a framework for understanding variability in creativity (Agnoli et al., 2019; Sánchez-Ruiz et al., 2011; Xu et al., 2019; Zenasni & Lubart, 2008). Nevertheless, findings are heterogeneous, partly because EI is not a unitary construct and because creativity can be assessed through different processes, domains, and levels of analysis.

A critical distinction is commonly drawn between ability EI, assessed through performance-based tasks, and trait EI, assessed through self-report questionnaires. In the present study, we adopt the trait EI framework. Trait EI should not be treated simply as a set of emotional competences or skills; rather, it refers to a constellation of emotion-related self-perceptions and dispositions located within the domain of personality and individual differences (Petrides et al., 2016; Petrides & Mavroveli, 2020). This positioning is theoretically important because trait EI captures how children typically perceive their emotional functioning, social relationships, self-regulation, and motivational tendencies, rather than their maximal performance on emotion-processing tasks. Trait EI has been associated with a broad range of adaptive outcomes, including well-being, social functioning, academic-related behavior, and school adjustment (Andrei et al., 2014; Bru-Luna et al., 2021; Perera, 2016; Sánchez-Álvarez et al., 2016; Özal et al., 2024). Recent evidence further indicates that trait EI can be reliably assessed in childhood using developmentally appropriate instruments such as the Trait Emotional Intelligence Questionnaire-Child Form (TEIQue-CF; Mavroveli et al., 2008; Russo et al., 2012).

The literature on EI and creativity suggests that the association may depend on both the EI model and the creativity outcome considered. In adult and young-adult samples, trait EI has been linked to creative personality, creative achievement, and domain-specific creative performance (Sánchez-Ruiz et al., 2010, 2011). A meta-analysis of the EI–creativity literature found a positive overall association, with stronger effects when EI and creativity were measured through self-report or trait-like indicators than when objective ability-based creativity tasks were used (Xu et al., 2019). More specifically, Giancola et al. (2024) reported that divergent thinking, but not convergent thinking, mediated the relationship between trait EI and real-world creative production in young adults, suggesting that trait EI may be especially relevant for exploratory idea generation. In children, studies have begun to show that EI-related resources may interact with creative training and contextual factors in predicting creative potential (Agnoli et al., 2022, 2023). However, most child research has either used global EI indicators or has not systematically distinguished between the divergent and convergent components of creative potential. This leaves open the question of which specific trait EI facets are most relevant for children’s creative thinking.

A facet-level approach is theoretically warranted. Creativity research has long emphasized the roles of intrinsic motivation, persistence, openness to novelty, and flexible adaptation in idea generation (Amabile, 1983; Hennessey & Amabile, 1998; Nijstad et al., 2010). These mechanisms map closely onto specific trait EI dimensions. Self-Motivation reflects children’s perceived capacity to initiate effort, persevere, and remain engaged in goal-directed activity; it may, therefore, support the sustained production of ideas in divergent thinking tasks. Adaptability reflects children’s perceived ability to adjust flexibly to new people, situations, and perspectives; it may, therefore, support the flexible restructuring of task stimuli, particularly when stimuli are abstract and do not immediately suggest familiar solutions. Conversely, convergent–integrative tasks may rely more strongly on selective combination, evaluative control, and task-specific representational skills, making them less directly dependent on emotion-related self-perceptions. Developmental timing may also matter as children progress through primary school, classroom norms, task familiarity, self-evaluative standards, and cognitive control change, potentially altering the role of trait EI facets in creativity.

### The Present Study

The present study addresses these gaps by providing a first exploration of the relationship between specific dimensions of trait EI and creative thinking abilities in primary school children aged 8 to 11 years. The study adds to previous work in three ways. First, it focuses on childhood, a developmental period in which both emotional self-perceptions and creative potential are still consolidating. Second, it examines trait EI at the facet level rather than relying only on a global score, allowing a more precise test of which emotional self-perceptions are associated with creative thinking. Third, it distinguishes divergent and convergent thinking across abstract and concrete graphical tasks using the Evaluation of Creative Potential (EPoC; Lubart et al., 2011). This design extends previous evidence on EI and creativity (Agnoli et al., 2022, 2023; Sánchez-Ruiz et al., 2011) by testing whether the link between trait EI and creativity is facet-specific, domain-sensitive, and developmentally moderated.

Based on this rationale, the present study addressed three research questions. The research questions were exploratory and descriptive, since no specific directional hypotheses can be formulated. Nonetheless, this study can provide the first evidence of possible associations between specific trait EI facets and creative potential, potentially shedding light on specific mechanisms relating emotional self-perceptions and creative behaviors during childhood.

Moreover, because prior findings indicate developmental fluctuations in creative potential during primary school, the analyses also explored whether grade moderated the possible associations between trait EI facets and creative potential. Again, no directional hypothesis was formulated for these moderation effects, given the limited facet-level developmental evidence available.

**RQ1.**  *How do divergent and convergent creative thinking abilities, as measured by the EPoC graphical tasks, manifest across grades in primary school children?*

**RQ2.**  *How are trait EI dimensions related to children’s divergent and convergent creative thinking abilities?*

**RQ3.**  *To what extent do specific trait EI dimensions predict children’s creative performance, and do these associations vary across grade levels?*

## 2. Materials and Methods

### 2.1. Participants and Procedure

The current study is part of a larger longitudinal project aimed at testing the impact of a tinkering training program on children’s creative abilities (Bevan et al., 2015; Petrich et al., 2013; Vossoughi & Bevan, 2014). The broader project used a pretest–posttest design with training and control groups. For the purposes of the present study, only pretest data were analyzed, and children from both the training and control groups were included before any intervention activities took place. Data were collected from 344 children (165 females; age range = 8–11 years), who were third-, fourth-, and fifth-grade students attending three public primary schools in Bologna, Italy (see Table 1). Recruitment was school-based and geographically circumscribed; therefore, the sample should be considered a convenience sample from an urban public-school context rather than a representative sample of the broader population.

Data collection took place in February 2025. Participation was voluntary and pseudonymized. All children who met the age and grade criteria and for whom parental consent was obtained were included in the study. All assessments were administered during regular school hours in the classroom, within whole-class groups, by trained researchers. Standardized instructions were provided orally. The study was conducted over two sessions, separated by approximately two weeks. During the first session, children completed the TEIQue-CF and the EPoC abstract tasks, including both divergent and convergent creativity assessments. During the second session, children completed the EPoC concrete tasks, again including both divergent and convergent components. For the EPoC tasks, children were given 10 min to complete the divergent thinking tasks and 15 min for the convergent thinking tasks. No fixed time limit was imposed for completing the TEIQue-CF. Due to special educational needs and language barriers, some children required individual support during the tasks; however, no major difficulties or disruptions were observed during administration. When necessary, administrators provided clarifications to ensure comprehension of the instructions, and a support teacher was present. In line with the inclusion-based model of the Italian public school system, children with special education needs are generally integrated into mainstream classrooms. Therefore, the participating classrooms may have included children with SEN, but the exact number could not be reported. The study was conducted in accordance with the Declaration of Helsinki and received approval from the Ethics Committee of the University of Bologna (protocol code 0390080). Written informed consent was obtained from the parents or legal guardians of all participating children prior to data collection.

### 2.2. Measures

The measures were administered in classroom settings by trained researchers, following standardized instructions and using the same order of tasks within each session.

#### 2.2.1. Trait Emotional Intelligence

Trait Emotional Intelligence (trait EI) was assessed using the Italian version of the Trait Emotional Intelligence Questionnaire-Child Form (TEIQue-CF), a self-report instrument developed for children aged 8–12 years within the trait EI theoretical framework (Mavroveli et al., 2008). The TEIQue-CF consists of 75 items rated on a 5-point Likert scale ranging from 1 (completely disagree) to 5 (completely agree). It provides a global trait EI score and scores on nine dimensions of children’s emotional self-perceptions: Adaptability, Affective Disposition, Emotion Expression, Emotion Perception, Emotion Regulation, Low Impulsivity, Peer Relations, Self-Esteem, and Self-Motivation. The original validation studies documented satisfactory internal consistency in independent child samples (Cronbach’s alpha = 0.76 and 0.73) and temporal stability, together with evidence of construct validity and relative independence from cognitive ability (Mavroveli et al., 2008). Evidence from Italian children and preadolescents supports the reliability and validity of the TEIQue-CF, including theoretically coherent associations with the Big Five and independence from cognitive ability (Russo et al., 2012). A recent systematic review further concluded that TEIQue child forms generally provide reliable and valid scores and are linked to relevant childhood outcomes, while also highlighting the need for more careful reporting of psychometric information in child samples (Özal et al., 2024). In the present study, the scale demonstrated good internal consistency (Cronbach’s α = 0.89). The subscale reliability coefficients ranged from low to acceptable, as follows: Adaptability (α = 0.68), Affective Disposition (α = 0.75), Emotion Expression (α = 0.62), Emotion Perception (α = 0.60), Emotion Regulation (α = 0.58), Low Impulsivity (α = 0.60), Peer Relations (α = 0.68), Self-Esteem (α = 0.71), and Self-Motivation (α = 0.64).

#### 2.2.2. Creativity: Visual Divergent and Convergent Thinking

Children’s creative potential was assessed using the graphical tasks of the Evaluation of Potential Creativity (EPoC) battery in Italian (Lubart et al., 2011). The EPoC is a multidimensional instrument designed to assess creative potential during development by distinguishing divergent–exploratory and convergent–integrative thinking across domains and task formats. Recent research with school-age children supports the EPoC as a structurally valid and developmentally appropriate instrument for profiling creative potential (Juriševič & Žerak, 2024). The present study employed only the graphical tasks, excluding verbal tasks in order to reduce the potential confounding role of age-related verbal proficiency and differences among children from non-Italian-speaking households.

For divergent thinking, children were asked to produce as many drawings as possible starting from a single visual stimulus that was either abstract or concrete. Divergent thinking performance was operationalized as *fluency*, measured by the total number of drawings produced. For convergent thinking, children were asked to produce one single drawing by integrating eight visual stimuli, again either abstract or concrete. Convergent thinking performance was evaluated in terms of the *originality* of the final drawing. The scoring procedure for convergent thinking involved a multi-step evaluation process based on the official EPoC scoring manual. First, a subset of 60 drawings was independently rated by three judges using the official EPoC scoring manual, assigning scores on a 7-point scale. After this initial rating, the judges discussed the evaluation criteria to clarify the shared scoring framework and adapt the manual to the present dataset. An interrater reliability analysis then yielded satisfactory agreement (ICC > 0.76), allowing the evaluation process to proceed. Subsequently, each judge independently scored approximately one-third of the full dataset (344 drawings × 2 tasks = 688 drawings), corresponding to approximately 230 drawings per judge, with an additional overlap of 30 drawings between judges. These overlapping evaluations were used to monitor interrater reliability throughout the scoring process, resulting in approximately 290 drawings evaluated per judge.

### 2.3. Data Analysis

Multiple regression analyses were conducted to examine the roles of age (operationalized as grade level) and trait EI dimensions in predicting creative performance during development. Specifically, children were grouped into three grade categories corresponding to the 3rd, 4th, and 5th grades, with mean ages of 9, 10, and 11 years, respectively. Building on prior findings indicating that creative abilities exhibit developmental changes during primary school (Alves-Oliveira et al., 2022; Claxton et al., 2005; Gralewski et al., 2016; Kim, 2011; Runco et al., 2017), as well as evidence that trait EI can be associated with creative performance in primary school children (Agnoli et al., 2022, 2023), three sets of variables were entered into the models to examine: (1) the effect of grade, (2) the effect of trait EI dimensions, and (3) the interaction between grade and trait EI dimensions on each creative potential index, in order to determine whether the influence of trait EI varied with age. The creative potential indices comprised fluency, assessed through divergent thinking (DT) tasks with abstract and concrete stimuli, and originality, assessed through convergent thinking (CT) tasks with abstract and concrete stimuli. All predictors and moderator variables were mean-centered prior to creating the interaction terms. Model parameters were estimated using a bootstrapping procedure with 1000 iterations, and the threshold for statistical significance was set at *p* < 0.05. Assumption checks informed us about the adequacy of these analyses: residuals appeared indeed as normally distributed and were independent in all models (Durbin-Watson between 1.68 and 1.93); moreover, multicollinearity was absent in all models (Tolerance between 0.44 and 0.94; VIF between 1.06 and 2.24). Moreover, using G*Power 3.1.9.7, we conducted a power analysis for multiple regressions with 19 predictors, defining a target effect size of f^2^ as 0.15 (assuming, therefore, a medium effect size, because of the lack of previous results), alpha level as 0.05, and statistical power as 0.95, which yielded a minimal sample size of 217 participants. Type I and II errors were fixed to the same value of 0.05 because both were considered equally important to avoid, and error rates of 0.05 were deemed tolerable for the present study. Analyses were performed using IBM SPSS Statistics (Version 26.0; IBM Corp., 2019).

## 3. Results

### 3.1. Descriptive Statistics

Descriptive statistics summarizing the performance of children from three grade levels on the four creative tasks are reported in Table 2.

**Figure 1:**
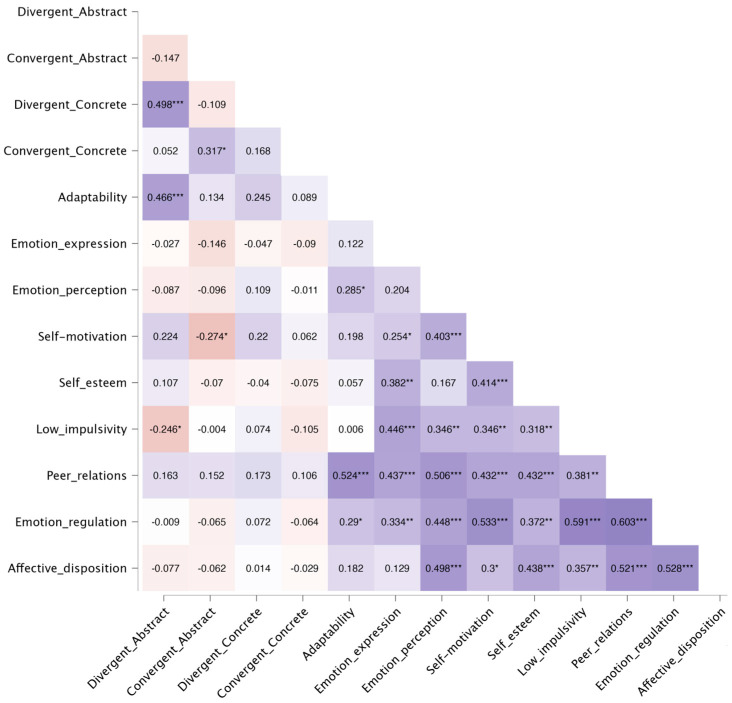
Heatmap of the correlations between creative potential abilities and trait EI in grade 3. Note. * *p* < 0.05; ** *p* < 0.01; *** *p* < 0.001.

**Figure 2:**
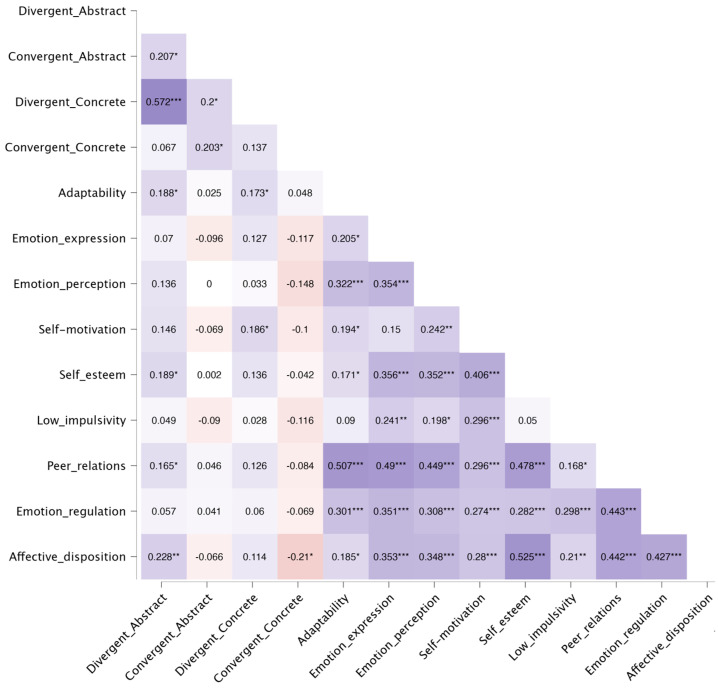
Heatmap of the correlations between creative potential abilities and trait EI in grade 4. Note. * *p* < 0.05; ** *p* < 0.01; *** *p* < 0.001.

**Figure 3:**
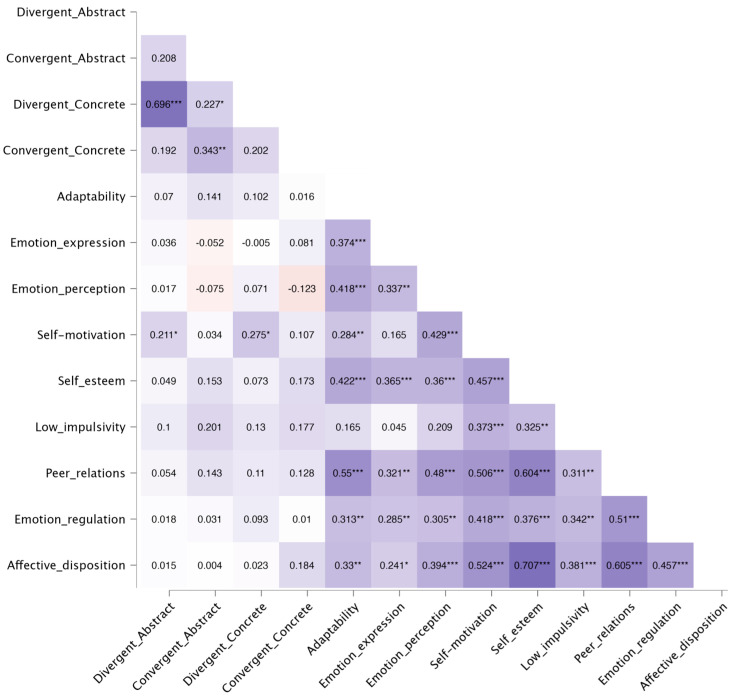
Heatmap of the correlations between creative potential abilities and trait EI in grade 5. Note. * *p* < 0.05; ** *p* < 0.01; *** *p* < 0.001.

Pearson correlations were conducted to examine the relationships between indices of creative potential and the nine dimensions of trait EI in children, as measured by the TEIQue-CF, across the three grades. As shown in Figure 1, Figure 2 and Figure 3, several trait EI dimensions were correlated with creative potential abilities, although the strength and pattern of these associations appeared to vary with age. Low impulsivity was negatively correlated with abstract DT abilities in the youngest group, whereas in fourth graders, Peer Relations and Affective Disposition were lowly positively associated with abstract DT abilities. In particular, Adaptability and Self-Motivation had a low-to-moderate association with DT abilities, with variations in these relationships observed across grade levels.

Adaptability concerns children’s self-perceptions of how well they adapt to a new situation and people (Mavroveli et al., 2008). For the youngest group (i.e., third graders), the Adaptability facet of trait EI showed the strongest correlation with abstract DT scores, as measured through fluency (*r* = 0.466, *p* < 0.001), on which they scored similarly to fifth graders (see Table 2). In fourth graders, Adaptability was significantly, though more modestly (*r* = 0.188, *p* < 0.05), correlated with abstract DT scores and, unlike in the other groups, also related to concrete DT scores (*r* = 0.173, *p* < 0.05).

Self-Motivation concerns children’s perception of their drive and effort (e.g., perceiving the initiative to perform better at school) (Mavroveli et al., 2008). In our sample, Self-Motivation was significantly correlated with both abstract (*r* = 0.211, *p* < 0.05) and concrete (*r* = 0.275, *p* < 0.05) DT tasks in fifth graders, although the correlations were small. In fourth graders, it was significantly, though modestly, correlated only with the concrete DT task (*r* = 0.186, *p* < 0.05). For the youngest group (third graders), Self-Motivation was negatively correlated with the abstract CT task (*r* = −0.274, *p* < 0.05).

### 3.2. The Role of Age and Trait EI as Predictors of Creative Potential

Multiple regression analyses were conducted to examine whether age and trait EI dimensions predicted children’s creative performance during development. While fluency, as measured through DT tasks with abstract stimuli, was not predicted by grade, it emerged to be predicted by two specific trait EI dimensions: Adaptability and Self-Motivation (Table 3).

Adding the block of interactions with grade in Step 3 slightly increased the explained variance (ΔR^2^ = 0.042), but this change was not statistically significant (ΔF = 1.516, *p* = 0.14), indicating that the interactions as a whole did not significantly improve the model. However, examination of individual predictors on the abstract DT task revealed a significant interaction between Adaptability and grade (β = −1.471, *p* < 0.05).

**Figure 4:**
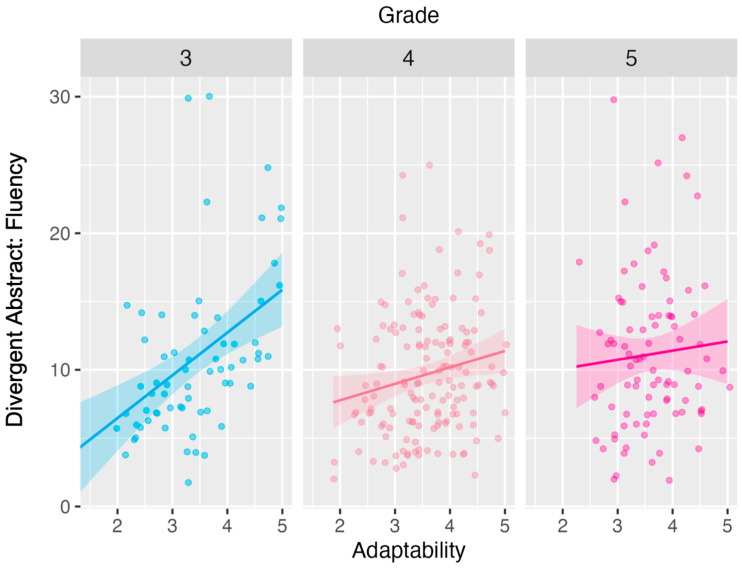
Change in the predictive role of Adaptability in abstract divergent thinking ability in relation to children’s grade.

This suggests that the effect of Adaptability on abstract DT ability varies across grades. A simple slope analysis, in particular, revealed that the increase in Adaptability was significantly associated with an increase in the abstract DT ability at grade 3 (b = 3.13, SE = 0.70, *p* < 0.01, 95% CI [1.74, 4.52]) and, even to a lesser extent, at grade 4 (b = 1.21, SE = 0.58, *p* = 0.04, 95% CI [0.60, 2.35]), but not at grade 5 (b = 0.67, SE = 0.91, *p* = 0.46, 95% CI [−1.12, 2.47]), as shown in Figure 4.

Consistent with earlier results, Adaptability (B = 1.008, *p* < 0.05) and Self-Motivation (B = 2.357, *p* < 0.01) again emerged as significant predictors of DT performance with concrete stimuli (Table 3). Increases in Adaptability and Self-Motivation were associated with higher DT performance, regardless of children’s grade.

**Figure 5:**
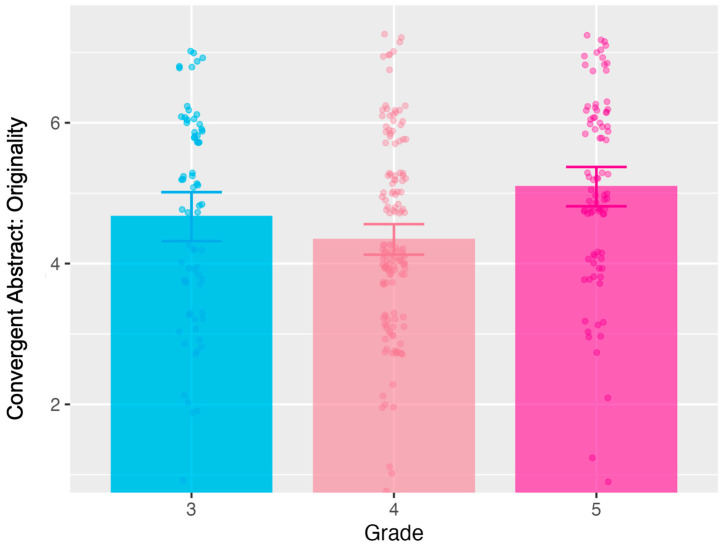
Change in concrete convergent ability with grade.

For CT with abstract stimuli, grade significantly predicted performance, indicating that this ability tendentially increases with age (Figure 5). Multivariate comparisons with Bonferroni correction indicated that performance was significantly lower at grade 4 compared to grade 5 (*p* < 0.01), suggesting a temporary dip in this ability at grade 4.

Finally, no significant predictors emerged in the regression model for CT with concrete stimuli, indicating that neither trait EI dimensions nor grade significantly predicted performance on this task (Table 4).

## 4. Discussion

The present study explored how specific trait EI dimensions are associated with children’s creative potential across the last three years of primary school. In response to RQ1, the descriptive and regression results indicated that creative performance varied across task type and grade, with a developmental pattern most visible for convergent thinking with abstract stimuli. In response to RQ2 and RQ3, the findings suggested that the association between trait EI and creativity was selective rather than global: Adaptability and Self-Motivation predicted divergent thinking, whereas no consistent trait EI predictors emerged for convergent thinking. These results support a facet-level and process-specific interpretation of the trait EI–creativity link in childhood.

The developmental pattern observed for convergent thinking should be interpreted cautiously. The abstract convergent task showed a grade-related effect, with a lower performance in grade 4 than in grade 5, consistent with research describing non-linear trajectories, temporary decreases, and subsequent rebounds in children’s creative abilities (Charles & Runco, 2001; Claxton et al., 2005; Gralewski et al., 2016; Kim, 2011). The present finding is compatible with the idea of a fourth-grade slump, but it does not establish a universal developmental decline. Rather, it suggests that the period around 9–10 years may be sensitive to changes in task demands, school expectations, self-evaluation, and representational strategies. This interpretation is also consistent with evidence that creativity development is multidimensional and may differ depending on whether tasks require fluency, originality, integration, abstract reasoning, or domain-specific knowledge (Barbot et al., 2015; Juriševič & Žerak, 2024).

The most robust pattern concerned divergent thinking. Adaptability positively predicted divergent thinking, particularly in the abstract task, and its effect varied across grades. The association was strongest in grade 3, weaker in grade 4, and no longer significant in grade 5. This pattern suggests that younger children who perceive themselves as more able to adapt to new situations may be better positioned to reinterpret abstract stimuli flexibly and generate multiple responses. As children grow older, other factors, such as accumulated task knowledge, executive control, classroom norms, or self-evaluative standards, may become more influential, thereby reducing the relative contribution of perceived adaptability. Self-Motivation, in contrast, showed a more stable association with divergent thinking. This is theoretically coherent because fluency tasks require sustained effort, persistence, and willingness to continue producing responses after the most obvious ideas have been exhausted. These findings align with motivational and dual-pathway models of creativity, which identify flexibility and persistence as two core routes to creative ideation (Agnoli et al., 2018; Amabile, 1983; Hennessey & Amabile, 1998; Nijstad et al., 2010).

The absence of consistent associations between trait EI facets and convergent thinking is also informative. Convergent–integrative tasks require children to construct a single original product by combining multiple elements. Although emotional self-perceptions may support engagement with the task, originality in this format may depend more strongly on representational integration, selective comparison, visual organization, and evaluative judgment. This interpretation is consistent with adult evidence suggesting that divergent thinking may be more proximal than convergent thinking in the pathway linking trait EI to creative production (Giancola et al., 2024). Thus, the present findings do not imply that trait EI is unrelated to creativity; rather, they suggest that trait EI facets may be more strongly involved in the exploratory generation of multiple ideas than in the integrative production of a single original response.

### 4.1. Theoretical and Practical Implications

Theoretically, this study contributes to a more differentiated understanding of the relationship between trait EI and creativity. First, it supports the value of treating trait EI as a personality-based individual-difference construct and examining its facets rather than relying only on global scores. Second, it suggests dimension-ability specificity: Adaptability was most relevant to abstract divergent thinking and developmentally sensitive, whereas Self-Motivation was more broadly related to divergent fluency. Third, the findings reinforce the distinction between divergent and convergent components of creative potential, showing that the emotional self-perceptions captured by trait EI do not necessarily predict all forms of creative performance equally.

From a practical perspective, the results should be read as preliminary indications rather than as evidence for direct intervention effects. The findings suggest that classroom contexts that encourage flexible exploration, tolerance of multiple possible answers, and sustained engagement may be compatible with the emotional self-perceptions that support divergent thinking. However, because trait EI is conceptualized within the personality domain, educational implications should not be framed as attempts to directly modify stable traits. A more cautious implication is that teachers can design learning environments that give children opportunities to experience adaptability and motivated engagement in creative tasks, for example, through open-ended problems, low-threat experimentation, constructive feedback, and activities that allow multiple solutions. Whether such environments influence trait EI, creativity, or their interplay must be tested in future intervention and longitudinal studies.

### 4.2. Limitations and Future Research Directions

Several limitations should be acknowledged. First, the study was exploratory and cross-sectional; therefore, the findings do not support causal claims or firm conclusions about developmental change. Second, the sample was drawn from three public primary schools in one Italian urban area and should not be considered representative of all children in this age range. Third, although the overall sample size was adequate for an exploratory school-based study, the number of predictors and interaction terms may reduce model stability, especially when analyses are considered separately across creative outcomes. Fourth, trait EI was assessed using self-report, which is appropriate within the trait EI framework but may be influenced by children’s self-understanding, reading comprehension, social desirability, or response style. Fifth, the study focused on graphical EPoC tasks only; this choice reduced the possible confounding role of verbal proficiency but limits conclusions about verbal, social, scientific, or other creative domains.

Future research should replicate these findings with larger and more diverse samples, report reliability and validity evidence for all scales in each sample, and use longitudinal designs to examine whether the roles of Adaptability and Self-Motivation change across development. Multi-informant approaches combining self-reports, teacher reports, behavioral observations, and performance-based measures could clarify how emotional self-perceptions relate to actual creative behavior in the classroom. Future studies should also examine contextual moderators, including classroom climate, teaching style, socioeconomic background, language status, and exposure to creative learning activities. Finally, intervention studies are needed to test whether learning environments designed to support flexible exploration, and sustained engagement can enhance divergent thinking and whether such changes are accompanied by shifts in children’s emotional self-perceptions.

## 5. Conclusions

In conclusion, the present study suggests that the relationship between trait EI and creative potential in childhood is selective, facet-specific, and developmentally sensitive. Adaptability and Self-Motivation were associated with children’s divergent thinking, whereas convergent thinking did not show comparable associations with trait EI facets. These results indicate that children’s emotional self-perceptions may be especially relevant for the exploratory generation of multiple ideas, rather than for all forms of creative performance. The main take-home message is that the trait EI–creativity link in childhood should be studied at the level of specific emotional self-perceptions, specific creative processes, and specific developmental periods.

## Figures and Tables

**Table 1:** Sample distribution across the three grades (3rd, 4th and 5th grade).

	3	4	5	Total
Males	41	86	52	179
Females	32	91	42	165
Total	73	177	94	344

**Table 2:** Descriptive statistics of the creative potential indexes at the three grades.

	Divergent Abstract			Convergent Abstract			Divergent Concrete			Convergent Concrete		
Grade	3	4	5	3	4	5	3	4	5	3	4	5
Mean	10.74	9.68	11.17	4.68	4.35	5.10	10.58	9.59	11.20	4.30	3.87	4.45
SD	5.79	4.53	5.65	1.50	1.33	1.35	5.95	4.64	6.73	1.46	1.41	1.35
Min.	2	2	2	1	1	1	0	1	2	1	1	1
Max.	30	25	30	7	7	7	30	30	30	7	7	7

**Table 3:** Hierarchical regression analyses examining the contributions of age (grade level), trait EI, and of their interactions on divergent thinking abilities.

	Divergent Abstract			Divergent Concrete		
	Step 1	Step 2	Step 3	Step 1	Step 2	Step 3
grade	0.265	0.227	0.087	0.402	0.217	0.190
adaptability		1.685 **	7.266 **		1.008 *	2.542
emotion expression		−0.003	−2.083		0.101	−0.265
emotion perception		−0.499	−2.802		−0.314	0.166
self-motivation		1.816 **	3.151		2.357 **	0.184
self-esteem		0.236	4.723		−0.561	−1.635
low impulsivity		−0.537	−5.443		0.074	0.795
peer relations		−0.119	1.256		0.439	2.424
emotion regulation		−0.682	−1.775		−0.318	−3.897
affective disposition		0.197	−1.542		−0.085	2.143
adaptability × grade			−1.471 *			−0.405
emotion exp. × grade			0.541			0.104
emotion perc. ×grade			0.612			−0.129
self-motivation. × grade			−0.353			0.549
self-esteem × grade			1.249			−0.157
low impulsivity × grade			−0.309			−0.481
peer relations × grade			0.283			0.890
emotion reg. × grade			0.450			−0.586
affective dis. × grade			−1.106			0.306
*R* ^2^	0.01	0.092	0.133	0.02	0.074	0.080
Δ*R*^2^		0.090 **	0.042		0.071 *	0.007
*F*	0.396	2.965 **	2.303 **	0.676	2.079 *	1.164
Δ*F*		3.247 **	1.516		2.231 *	0.210
*df*	303	294	285	271	262	253

Note: * *p* < 0.05; ** *p* < 0.01.

**Table 4:** Hierarchical regression analyses examining the contributions of age (grade level) and trait EI to convergent thinking abilities.

	Convergent Abstract			Convergent Concrete		
	Step 1	Step 2	Step 3	Step 1	Step 2	Step 3
grade	0.224 *	0.252 *	0.248 *	0.045	0.100	0.141
adaptability		0.076	−0.345		0.109	−0.062
emotion expression		−0.363 *	−1.273		−0.094	−1.335
emotion perception		−0.079	0.188		−0.289	1.059
self-motivation		−0.315	−1.912 *		0.080	0.010
self-esteem		0.124	−0.304		0.087	−0.141
low impulsivity		0.116	−0.166		0.014	−0.844
peer relations		0.550 *	2.529		0.181	0.760
emotion regulation		0.042	−0.333		−0.084	0.096
affective disposition		−0.252	−0.373		−0.149	−1.273
adaptability × grade			0.100			0.031
emotion exp. × grade			0.222			0.306
emotion perc. × grade			−0.067			−0.332
self-motivation. × grade			0.390			0.009
self-esteem × grade			0.076			0.214
low impulsivity × grade			−0.484			−0.140
peer relations × grade			0.102			−0.032
emotion reg. × grade			0.017			0.269
affective dis. × grade			0.114			0.061
*R* ^2^	0.013	0.067	0.092	0.01	0.025	0.054
Δ*R*^2^		0.54	0.25		0.025	0.028
*F*	3.946 *	2.071 *	1.488	0.145	0.675	0.745
Δ*F*		1.851	0.851		0.733	0.827
*df*	298	289	280	268	259	250

Note: * *p* < 0.05.

## Data Availability

The data presented in this study are openly available in the OSF repository at https://osf.io/63q57/overview (accessed on 13 April 2026).

## Abbreviations

The following abbreviations are used in this manuscript:

CT	Convergent Thinking
DT	Divergent Thinking
EPoC	Evaluation of Creative Potential
Trait EI	Trait Emotional Intelligence
TEIQue-CF	Trait Emotional Intelligence Child Form
